# Antioxidants and Calcium Modulators Preclude in Vitro Hepatitis B Virus–Induced Mitochondrial Damage

**DOI:** 10.5152/tjg.2023.21290

**Published:** 2023-10-01

**Authors:** Kehkshan Jabeen, Aneela Javed, Sobia Manzoor, Shaheen Shahzad

**Affiliations:** 1 Department of Biological Sciences, Genomics Research Lab, International Islamic University Islamabad, Islamabad, Pakistan; 2 Healthcare Biotechnology, Atta-ur Rahman School of Applied Biosciences (ASAB), National University of Science and Technology, Islamabad, Pakistan

**Keywords:** Antioxidant, calcium chelators, hepatitis B virus, mitochondrial dynamics, reactive oxygen species

## Abstract

**Background/Aims:**

Hepatitis B virus induces mitochondrial damage via the production of reactive oxygen species and concomitant with deregulation of calcium homeostasis. The current study evaluates the potential of antioxidant and calcium modulators for inhibition of hepatitis B virus-induced mitochondrial damage using* in vitro* cell culture models.

**Materials and Methods:**

Hepatitis B virus-induced mitochondrial fragmentation was observed by immunofluorescence confocal microscopy in hepatitis B virus-infected cell lines (HepG2 and HepAD38). Differential protein expression of mitochondrial fragmentation markers, dynamin-related protein 1 and phospho-dynamin-related protein 1, were evaluated both pre- and posttreatment with antioxidant *N*-acetyl-l-cysteine and calcium modulators like 1,2-bis(2-aminophenoxy)ethane-*N,N,N′,N′*-tetraacetic acid tetrakisacetoxymethyl ester, ethylene-bis (oxyethylenenitrilo) tetraacetic acid glycol ether diamine tetraacetic acid-acetoxymethyl ester, and ruthenium amine complex by western blot analysis.

**Results:**

A slight reduction in mitochondrial fragmentation in both cell lines was observed post-antioxidant treatment with a partial prevention observed with calcium modulators. The expression of phospho-dynamin-related protein 1 was significantly upregulated (*P = .*0007, *P* = .003) in both hepatitis B virus-infected cell lines compared to uninfected cells. In line with these observations, the expression of dynamin-related protein 1 and phospho-dynamin-related protein 1 was found to be significantly downregulated with *N*-acetyl-l-cysteine treatment in both cell lines (*P = .*003, *P* = .002), respectively. A nonsignificant trend was observed in the case of calcium modulators treatment.

**Conclusions:**

Current study indicates that the mitochondrial fragmentation induced by hepatitis B virus infection can be reduced after antioxidant treatment pointing toward exploring better drug targets for the prevention of hepatitis B virus-induced mitochondrial fragmentation and associated liver damage.

**Main Points**

- The potential of antioxidant and calcium modulators for inhibition of hepatitis B virus (HBV)-induced mitochondrial damage was evaluated by using *in vitro* cell culture models.
- Hepatitis B virus-induced mitochondrial damage was observed *in vitro* via confocal microscopy as well as elevated levels of mitochondrial fragmentation markers dynamin-related protein 1 (DRP1) and phospho-DRP1 (Ser616) by western blot analysis.
- Inhibition of HBV-induced mitochondrial damage in HBV-expressing cell lines was observed by posttreatment with antioxidant (*N*-acetyl-l-cysteine (NAC) and calcium blockers ethylene-bis (oxyethylenenitrilo) tetraacetic acid, 1,2-bis (2-aminophenoxy) ethane-*N,N,N′,N′*-tetraacetic acid tetrakisacetoxymethyl ester, ruthenium amine complex both via confocal microscopy and downregulation of DRP1 and phospho-DRP1 (Ser616) by western blot analysis.
- This study indicates that both NAC and calcium modulators have partially prevented mitochondrial fragmentation as well as downregulated the levels of mitochondrial fission protein; however, this partial prevention was slightly better seen in the case of antioxidant (NAC) as compared to calcium modulators.

## Introduction

Hepatitis B virus is the most virulent agent of chronic infection and holds a major health burden all over the world. Hepatitis B virus has affected 257 million people in the world so far.^1^ It is linked with life-threatening end-stage liver damage. Hepatitis B virus infection alters the cellular immune response to establish persistent infection.^2^

Hepatitis B virus expression is linked with mitochondrial damage along with oxidative stress in the progression of liver impairment.^3-5^ Three HBV-encoded proteins, multifunctional X protein named as hepatitis B virus X protein (HBx) hepatitis surface antigen (HBsAg), and core (HBcAg) antigens, are also involved in interacting with mitochondria and ultimately induce oxidative stress. HBx is a regulatory protein that is required for the viral life cycle both *in vitro* and *in vivo* and has an intricate part in the diverse cellular and physiological processes.^6^ It is reported to take part in a vital role during HBV progression. The expression of HBx leads to varying mitochondrial transmembrane potential (ΔΨm), lacking cristae with swollen mitochondria and increase in reactive oxygen species (ROS) level which results in deregulation of calcium level in mitochondria disrupting the homeostasis and ultimately leading to mitochondrial dysfunction.^7,8^ Furthermore, HBsAg along with HBcAg variants assemble into the endoplasmic reticulum (ER) appropriately in infected cells also provoke ER stress with unfolded protein response (UPR). It provokes the discharge of free calcium ions into the cytoplasm by following induction of oxidative stress.^9-11^

Like other infections, HBV alters calcium (Ca^2+^) signaling to make a suitable cellular environment for viral replication,^12^ restrains an antiviral immune reaction segment, furthermore controls cell signaling cascades.^13^ According to prior researches, in several established cell lines, the HBx expands the basal cytosolic Ca^2+^,^14,15^ which is required at some levels during HBV replication in these cells, that also counts for capsid assembly, HBV polymerase initiation, as well as for HBV genome replication.^8,16-18^ Modified Ca^2+^ signaling along with raised Ca^2+^ have been observed in HBV reproducing cells. These perceptions offer help for the idea that viruses may alter cell Ca^2+^ signaling pathways for purpose of their determination and resulting pathogenesis. Virally moderated cell Ca^2+^ signaling pathways could legitimately impact the improvement of viral-related illnesses.^12^

Mitochondria tend to undertake 2 contrasting events of fission and fusion. Dynamin-related protein 1 (DRP1), the mitochondrial fission protein, is essential for the course of mitochondrial fission. The interaction of (Fis-1) (fission related protein-1) and MFF (mitochondrial fission factor) leads to the production of small punctuated mitochondria.^19-21^ For its translocation to mitochondria, post-translational modifications of DRP1 on Ser 616 (S616) via cyclin-dependent kinase (CDK) 1/cyclin B or CDK5 is important. Thus, it overall promotes mitochondrial fission during mitosis.^22,23^

In our previous study scavenger of ROS, inhibitor and chelators of calcium have been used,^24^ as well as described in different reports. For example, report affirms that antioxidant (*N*-acetyl-l-cysteine (NAC)) impedes oxygen free radical effects.^25,26^ Similarly, as a result of using calcium modulators, blockage of calcium signals may have occurred. By using 1,2-bis(2-aminophenoxy)ethane-*N,N,N′,N′*-tetraacetic acid tetrakisacetoxymethyl ester (BAPTA-AM) or ethylene-bis (oxyethylenenitrilo) tetraacetic acid glycol ether diamine tetraacetic acid-acetoxymethyl ester (EGTA-AM), intercellular [Ca^2+^] chelation can be obtained. Whereas, using ruthenium amine complex (Ru360) the mitochondrial calcium uptake may be inhibited. As debated previously, HBV replication needs augmented cytosolic calcium level. This is successfully attained through HBV infection by means of shifting the mitochondrial Ca^2+^ uptake. Mitochondria are regulators of cellular calcium signaling. Though, no decisive study describes contradiction of the detrimental consequences of ROS and calcium deregulation in association of prevention of HBV-induced mitochondrial damage by reducing the ROS and restoration of the calcium homeostasis.

In the current study, we investigated possibility of prevention of HBV-induced mitochondrial damage by reducing the ROS and restoring the calcium homeostasis with the treatment of antioxidant (NAC) and calcium modulators BAPTA-AM, EGTA-AM, and Ru360.

## Materials and Methods

### Cells Lines and Plasmids

Procuring the HepG2 human hepatoma cell line was made possible from the American Type Culture Collection and cells were maintained as described previously.^24^ Dr. Jing-James Ou (University of Southern California) contributed generously pHBV1.3mer DNA encoding wild-type HBV genome. Dr. Christoph Seeger (Philadelphia, Pa, USA)^27^ kindly supplied HepAD38 cell line.^27^ HepAD38 cell line anchorage the entire HBV genome under tetracycline-responsible promoter which was retained as reported earlier.^24,27^ HepG2 cell line was grown into 6-well plate and following manufacture protocol transiently transfected with the plasmid (pHBV1.3mer) (300 ng) encoding 1.3 merHBVgenome along with TransIT^®^-LT1 transfection reagent (Mirus; Madison, Wis, USA). HepG2 and HepAD38 cell lines were grown with and without treatment with NAC (Millipore Sigma, Mo, USA), BAPTA-AM (Abcam: Cambridge, Mass, USA), EGTA-AM (Calbiochem, Calif, USA), and Ru360 (EMD Millipore Corp; Billerica, Mass, USA) that block Ca^+2^ uptake into mitochondria within HepAD38 as well as HepG2/pHBV1.3 cell lines, respectively, reported previously.^24^

### Antibodies

For western blot DRP1 (D6C7) rabbit mAb #8570 (Cell Signaling, Calif, USA) 1 : 1000 dilutions, for phospho-DRP1 (Ser616) antibody #3455 (Cell Signaling) 1:1000 dilutions, and for rabbit polyclonal GAPDH antibody FL-335 (Santa Cruz, Calif, USA) 1:1000 dilutions were used. However, 1:10 000 dilutions were used for mouse-anti rabbit IgG HRP-conjugated Sc-2357 (Santa Cruz, Calif, USA) and anti-mouse IgG HRP-conjugated (Santa Cruz, Calif, USA). For immunofluorescence, rabbit HBcAg polyclonal antibody (Santa Cruz, Calif, USA), 1:300 dilutions and anti-rabbit 488 Alexa Fluor (Invitrogen, San Diego, Calif, USA), 1:600 dilutions were used.

### Immunofluorescence Analysis

Immunofluorescence confocal microscopy was performed by growing HepG2/pHBV1.3 and HepAD38 cells on coverslips. For immunofluorescence before cells fixation, incubation of HepAD38 and HepG2/pHBV1.3 cells was done with 50 ηM MitoTracker Red (Invitrogen, Calif, USA) for 30 minutes at 37°C. After removal of medium from plates, cells were fixed by incubating in 4% paraformaldehyde at room temperature for 10 minutes. After fixation, cells were washed 5 times with 1× phosphate-buffered saline (PBS). Next, cells were blocked with blocking buffer (0.1% TritonX100 + 10% bovine serum albumin [BSA] + 10% FBS) at room temperature in 1× PBS for 2 hours. After blocking, cells were probed with rabbit HBcAg polyclonal antibody overnight at 4°C. Further, 3 times washing of cells was made possible with 1× PBS and then incubated with secondary antibody in buffer B (10% BSA + 10% FBS) in PBS for 2 hours at room temperature. After secondary antibody incubation, cells were washed thrice with PBS. Further, Reagent ProLong® Gold Antifade (Invitrogen) was used for mounting with diamidino-2-phenylindole (DAPI) to stain the nuclei. An Olympus FluoView 1000 confocal microscope was used to visualize the images under a 100× oil objective

### Immunoblotting

In the previous study, we reported that HepAD38 cells after 3 days post-HBV induction and HepG2 cells after 5 days post-HBV induction whole cell lysate for Sodium Dodecyl Sulphate-Polyacrylamide Gel Electrophoresis (SDS PAGE) was prepared. Infected cells were treated with 250 μm NAC, 5 μm EGTA-AM, and BAPTA-AM at 24 hours and 10 μm Ru360 at 12 hours during transfection. The cells were harvested after attaining 70% confluency and then further preceded for protein quantification, followed by further use in SDS-gel electrophoresis and western blot assay. Immunoblotting was performed as described in previous report.^24^

### Statistical Analysis

All the experiments were performed in triplicates (±SD) and significance was calculated by Student’s *t*-test (^*^ *P* < .05, ^**^ *P* < .01, ^***^ *P* < .001) by using Graph-Pad Prism 5.01 software (GraphPad Software, Inc., San Diego, Calif, USA).

## Results

## Prevention of Mitochondrial Fragmentation by N*-*Acetyl-l-Cysteine Treatment

**Figure 1.**
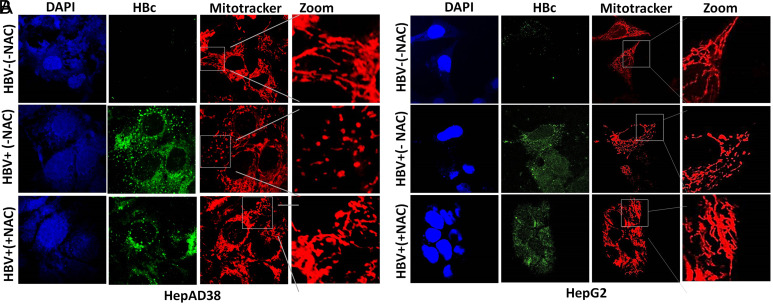
Effect of NAC treatment on HBV-induced mitochondrial fragmentation. (A) HBV replicating cells (HepAD38 HBV on (+) and HBV off (−) cells) (B) (HepG2 transfected (+) and untransfected (−) cells) HepG2 cells transiently transfected with pHBV1.3 (300 ng) encoding HBV genome. At 36 hours post-HBV induction, cells were treated with 250 μM NAC for 24 hours and stained with MitoTracker. The cells positive for HBV transfection were immunostained with anti-HBc, while nuclei were stained with DAPI. MitoTracker (red), HBc (green), and DAPI (blue) were marked. Zoomed insets showed typical tubular and non-fragmented mitochondria in HBV−(−NAC) panel. Clear mitochondrial fragmentation was seen in HBV+(−NAC) cells. HBV+(+NAC) cells were showing partial prevention of mitochondrial fragmentation after treatment with NAC in both the cell lines. HBV, Hepatitis B virus; NAC, *N*-acetyl-l-cysteine.

The results illustrated slight mitochondrial fragmentation within both HBV-infected cell lines HepG2/pHBV1.3 and HepAD38 (Figure 1A and 1B as compared to non-infected controls HBV−(−NAC) where normal tubular mitochondria were observed under confocal microscope). However, in both cell lines after treatment with antioxidant (NAC), the extent of mitochondrial fragmentation was considerably reduced. As can be seen clearly in HBV+(+NAC) panels, tubular and less fragmented mitochondria are visible as compared to fully fragmented mitochondria in the case of untreated cells (Figure 1A and 1B).

### Upregulation in Phospho-Dynamin-Related Protein 1 (Ser616) Expression after Hepatitis B Virus Infection

**Figure 2.**
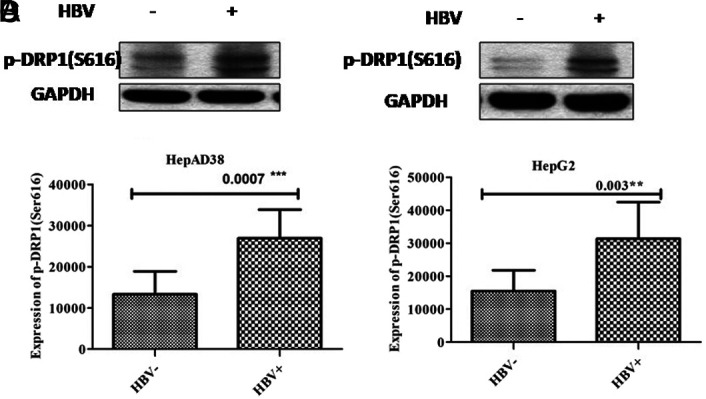
Upregulation of p-DRP1 (Ser616) expression post-HBV infection. The protein was extracted from HepG2/pHBV1.3 (300 ng) and HepAD38 cells 5 and 3 days post-HBV induction, respectively. As an internal control, GAPDH was used. Phospo-DRP1 expression was significantly upregulated in infected cells in comparison to uninfected cells. For statistical analysis, Student’s *t*-test was used. HBV+ represents HBV positive group and HBV− represents HBV negative group. DRP1, phospho-dynamin-related protein 1; p-DRP1, phospho-dynamin-related protein 1; HBV, hepatitis B virus.

The findings showed that in both cell lines, p-DRP1 expression in infected cells (*P* = .0007, *P* = .003) was considerably upregulated in contrast with uninfected cells as illustrated in Figure 2A and 2B, respectively.

## Phospho-Dynamin-Related Protein 1 Downregulation after Treatment with Antioxidant (N*-*Acetyl-l-Cysteine)

**Figure 3.**
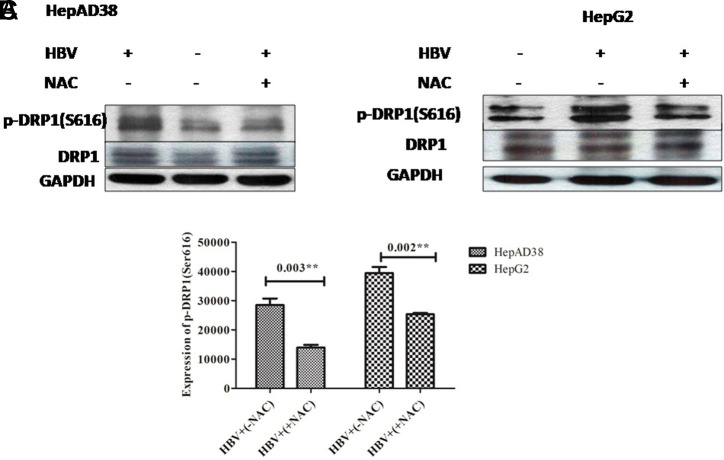
The effect of NAC treatment on the expression of p-DRP1 (Ser616) in HBV (A) HepAD38 (B) HepG2/pHBV1.3 cell lines. Immunoblots of extracted cell lysates from (A) HepAD38 cells 3 days post-induction of HBV gene expression and (B) HepG2/pHBV1.3 (300 ng) encoding wild-type HBV genome cell line 5 days post-HBV induction. As an internal loading control, GAPDH was used. Phospho-DRP1 (Ser616) downregulation was observed in HBV+ cells treated with 250 μM NAC for 24 hours. In both infected cell lines, significant differences were identified with *P = .*003^**^ and 0.002^**^, respectively. The X-axis shows the different groups and the Y-axis specifies the protein expression values of p-DRP1 (Ser616) in both cell lines. (C) Student’s *t*-test was used for analysis. The level of p-DRP1 (Ser616) protein expression was downregulated as quantified by ImageJ software. DRP1, phospho-dynamin-related protein 1; p-DRP1, phospho-dynamin-related protein 1; HBV, hepatitis B virus; NAC, *N*-acetyl-l-cysteine.

Dynamin-related protein 1 has an impact on mitochondrial morphology and has significance in mitochondrial and peroxisomal fission in mammalian cells too.^6,21,23,28,29^ Hepatitis B virus provokes the expression and phosphorylation (Ser616) of DRP1 as reported previously.^5^ After DRP1-dependent mitochondrial fission is altered directly or indirectly, apoptosis can be altered through various signaling pathways. We therefore explored whether NAC treatment can affect the expression of DRP1 and phosphorylation of p-DRP1 (Ser616). The expression pattern of DRP1 and p-DRP1 (Ser616) in HepG2/pHBV1.3 and HepAD38 cell lines was investigated with or without treatment of NAC. It had been noticed that antioxidant (NAC) treatment notably downregulated the phosphorylation (Ser616) of Drp1 (*P = .*003, *P* = .002) within both infected cell lines respectively as illustrated in Figure 3A -C, HBV+(+NAC).

**Partial Prevention of Mitochondrial Fission After Treatment with Calcium Modulators**

**Figure 4.**
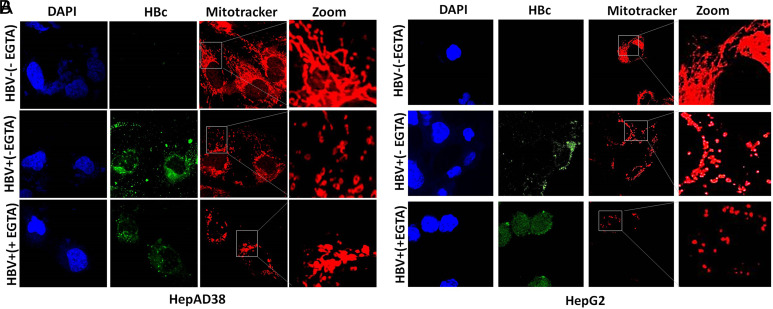
Effect of EGTA-AM on mitochondrial fragmentation. (A) HBV replicating cells (HepAD38) HBV on (+) and HBV off (−) cells) (B) (HepG2 transfected (+) and untransfected (−) cells) HepG2 cells were transiently transfected with plasmid (300 ng) encoding 1.3 mer HBV genome. At 36 hours post-HBV induction, cells were treated with 5 μM EGTA-AM for 24 hours and stained with MitoTracker. The cells positive for HBV transfection were immunostained with anti-HBc while nuclei were stained with DAPI. MitoTracker (red), HBc (green), and DAPI (blue) were marked. Zoomed insets showed that no fragmented mitochondria (typical tubular mitochondria) were seen in HBV−(−EGTA) panel. Clear mitochondrial fragmentation was seen in HBV+(−EGTA) panel. Instead of mitochondrial fragmentation, a mere swelling of mitochondria was observed in HBV+(+EGTA) panel treated with 5 μM EGTA-AM for 24 hours in both infected cell lines. BAPTA-AM, 1,2-bis(2-aminophenoxy)ethane-*N,N,N′,N′*-tetraacetic acid tetrakisacetoxymethyl ester; EGTA-AM, ethylene-bis (oxyethylenenitrilo) tetraacetic acid Glycol ether diamine tetraacetic acid-acetoxymethyl ester; HBV, hepatitis B virus.

**Figure 5.**
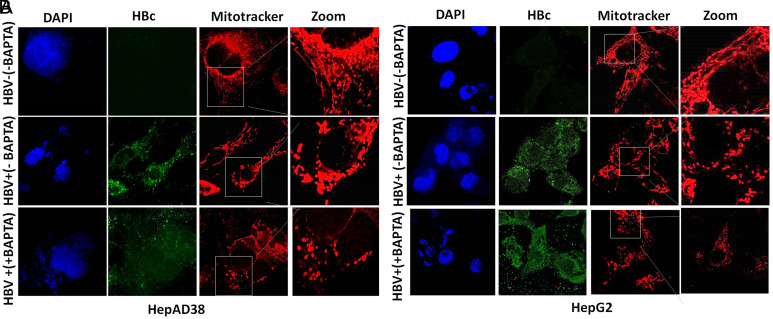
Effect of BAPTA-AM treatment on HBV-induced mitochondrial fragmentation. (A) HBV replicating cells (HepAD38. HBV on (+) and HBV off (−) cells) (B) (HepG2 transfected (+) and untransfected (–) cells) HepG2 cells were transiently transfected with Plasmid (300 ng) encoding 1.3mer HBV genome. At 36 hours, post-HBV induction cells were treated with 5 μM BAPTA-AM for 24 hours stained with MitoTracker. The cells positive for HBV transfection were immunostained with anti-HBc while nuclei were stained with DAPI. MitoTracker (Red), HBc (green), and DAPI (blue) were marked. Zoomed insets showed that no fragmented mitochondria (typical tubular mitochondria) were seen in HBV−(−BAPTA) panel. Instead of mitochondrial fragmentation, swelling of mitochondria was observed in HBV+(+ BAPTA) panel treated with 5 μM BAPTA-AM for 24 hours in both HepG2/pHBV1.3 and HepAD38 cell lines. BAPTA-AM, 1,2-bis(2-aminophenoxy)ethane-*N,N,N′,N′*-tetraacetic acid tetrakisacetoxymethyl ester; HBV, hepatitis B virus.

**Figure 6.**
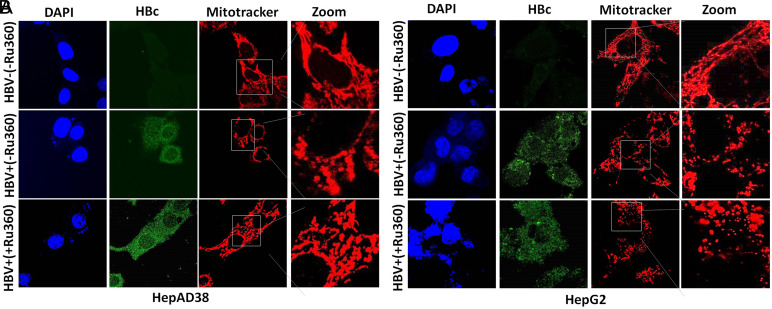
Effect of Ru360 treatment on mitochondrial fragmentation. (A) HBV replicating cells (HepAD38 (HBV on (+) and HBV off (−) cells) (B) (HepG2 transfected (+) and untransfected (−) cells) HepG2 cells were transiently transfected with Plasmid (300 ng) encoding 1.3 mer HBV genome. At 36 hours, post-HBV induction cells were treated with 10 μM Ru360 for 12 hours stained with MitoTracker. The cells positive for HBV transfection were immunostained with anti-HBc while Nuclei were stained with DAPI. MitoTracker (red), HBc (green), and DAPI (blue) were marked. Zoomed insets showed that no fragmented mitochondria (typical tubular mitochondria) were seen in HBV−(−Ru360) panel. Clear mitochondrial fragmentation was seen in HBV+(−Ru360) panel. Instead of mitochondrial fragmentation, a mere swelling of mitochondria was observed in HBV+(+Ru360) panel treated with 10 μM Ru360 for 12 hours in both HepAD38 and HepG2/pHBV1.3 cell lines. HBV, hepatitis B virus; p-DRP1, phospho-dynamin-related protein 1; Ru360, ruthenium amine complex.

The expression of HBV is associated with deregulation in calcium homeostasis leading toward mitochondrial damage and fission.^7,8,30^ The mitochondrial fragmentation was observed by confocal microscopy in both HBV-infected cell lines (HepG2/pHBV1.3 and HepAD38). Conversely, this mitochondrial damage was prevented after treatment of BAPTA-AM, EGTA-AM, and Ru360 in HepG2/pHBV1.3 and HepAD38 cell lines respectively. Instead of mitochondrial fragmentation, a mere swelling of mitochondria was observed in BAPTA-AM-, EGTA-AM-, and Ru360-treated cells in contrast with untreated cells where fragmented and fissioned mitochondrions are clearly visible (Figures 4A, 4B, 5A, 5B, 6A and 6B in HBV+(+EGTA), HBV+(+BAPTA), and HBV+(+Ru360) panels). These results revealed that a partial prevention of mitochondrial fragmentation caused by HBV infection can be achieved by regulating the calcium homeostasis in vitro.

**Downregulation of Phospho-Dynamin-Related Protein 1 (Ser616) by Treatment with Calcium Modulators**

**Figure 7.**
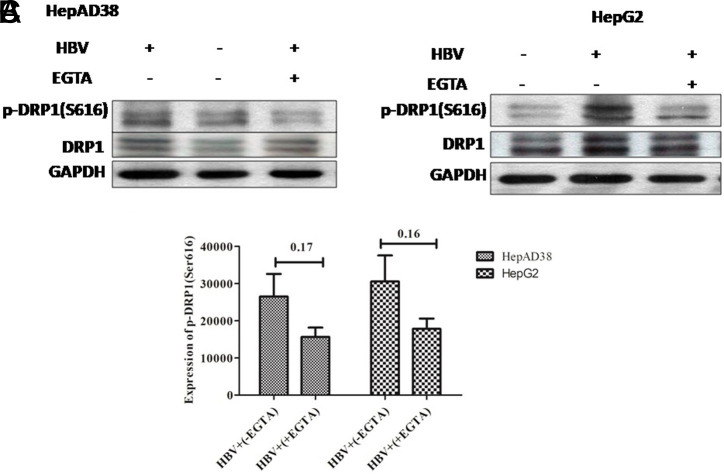
The effect of EGTA-AM treatment on the expression of p-DRP1 (Ser616) in HBV (A) HepAD38 (B) HepG2/pHBV1.3 cell lines. Immunoblots of extracted cell lysates from (A) HepAD38 cells 3 days post-HBV induction (B) HepG2/pHBV1.3 (300 ng) encoding wild-type HBV genome cell line 5 days post-HBV induction. As an internal loading control, GAPDH was used. Down-regulation of p-DRP1 (Ser616) was seen in HBV+ cells treated with 5 μM EGTA-AM. Significant differences were not seen in both HepAD38 and HepG2/pHBV1.3 cell lines with P = .17 and .16, respectively. The X-axis indicates the different groups and the Y-axis shows the protein expression values of p-DRP1 (Ser616) in both cell lines. (C) Student’s t-test was used for analysis. The level of p-DRP1 (Ser616) protein expression was downregulated as quantified by ImageJ software. BAPTA-AM, 1,2-bis(2-aminophenoxy)ethane-N,N,N′,N′-tetraacetic acid tetrakisacetoxymethyl ester; DRP1, phospho-dynamin-related protein 1; p-DRP1, phospho-dynamin-related protein 1; EGTA-AM, ethylene-bis (oxyethylenenitrilo) tetraacetic acid Glycol ether diamine tetraacetic acid-acetoxymethyl ester; HBV, hepatitis B virus.C).

**Figure 8.**
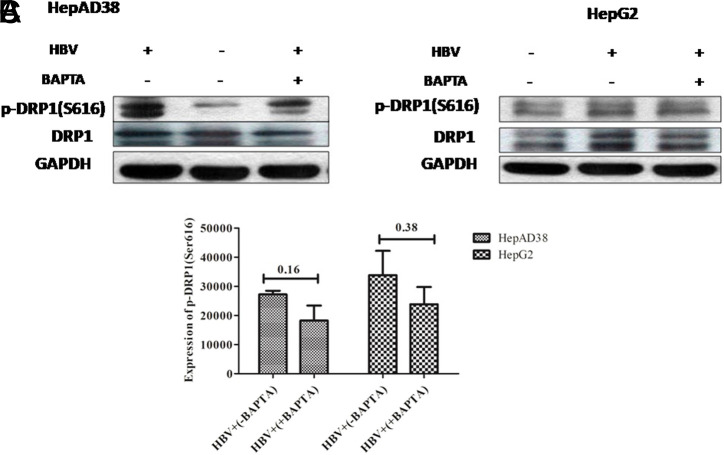
Effect of BAPTA-AM treatment on the expression of p-DRP1 (Ser616) in HBV (A) HepAD38 (B) HepG2/pHBV1.3 cell lines. Immunoblots of extracted cell lysates from (A) HepAD38 cells 3 days post-HBV induction (B) HepG2/pHBV1.3 (300 ng) encoding wild-type HBV genome cell line 5 days post-HBV induction. As an internal loading control, GAPDH was used. Downregulation of p-DRP1 (Ser616) was seen in HBV+ cells treated with 5 μM BAPTA-AM. Significant differences were not seen in both HepAD38 and HepG2/pHBV1.3 cell lines with *P = .*16 and .38, respectively. The X-axis indicates the different groups and the Y-axis shows the protein expression values of p-DRP1 (Ser616) in both HepAD38 and HepG2/pHBV1.3 cell lines. (C) Student’s *t*-test was used for analysis. The level of p-DRP1 (Ser616) protein expression was downregulated as quantified by ImageJ software. BAPTA-AM, 1,2-bis(2-aminophenoxy)ethane-*N,N,N′,N′*-tetraacetic acid tetrakisacetoxymethyl ester; DRP1, phospho-dynamin-related protein 1; p-DRP1, phospho-dynamin-related protein 1; HBV, hepatitis B virus.

**Figure 9.**
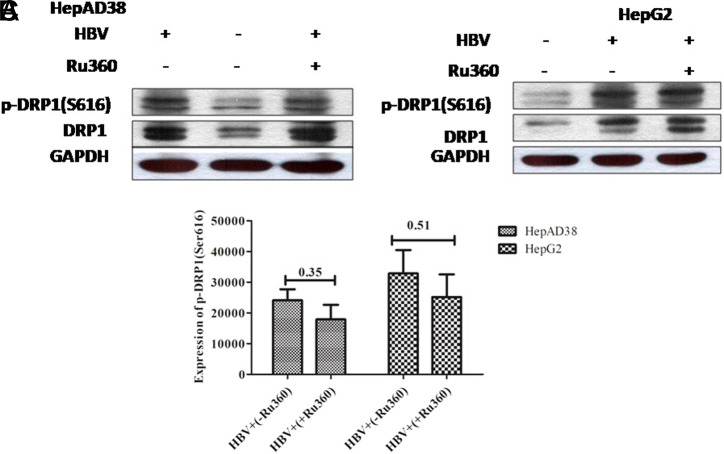
Effect of Ru360 treatment on the expression of p-DRP1 (Ser616) in HBV (A) HepAD38 (B) HepG2/pHBV1.3 cell lines. Immunoblots of extracted cell lysates from (A) HepAD38 cells 3 days post-HBV induction (B) HepG2/pHBV1.3 (300 ng) encoding wild-type HBV genome cell line 5 days post-HBV induction. As an internal loading control, GAPDH was used. Downregulation of p-DRP1 (Ser616) was seen in HBV+ cells treated with 10 μM Ru360 for 12 hours. Significant differences were not seen in both HepAD38 and HepG2/pHBV1.3 cell lines with *P = .*35 and 0.51, respectively. The X-axis indicates the different groups and the Y-axis shows the protein expression values of p-DRP1 (Ser616) in both HepAD38 and HepG2/pHBV1.3 cell lines. (C) Student’s *t*-test was used for analysis. The level of p-DRP1 (Ser616) protein expression was downregulated as quantified by ImageJ software. HBV, hepatitis B virus; p-DRP1, phospho-dynamin-related protein 1; Ru360, ruthenium amine complex.

The effect of EGTA-AM, BAPTA-AM, and Ru360 on the expression of mitochondrial fission markers, DRP1 and p-DRP1 (Ser616), by western blot was analyzed. Results revealed that there was a rise of DRP1 and p-DRP1 (Ser616) expression in HepG2/pHBV1.3 and HepAD38 cell lines post-HBV infection. However, after the treatment of calcium modulators (EGTA-AM, BAPTA-AM, and Ru360), a downregulation in the p-DRP1 (Ser616) expression was observed (*P = .*17, *P* = .16) (*P = .*16, *P* = .38) and (*P = .*35, *P* = .51) in both infected cell lines respectively. Nevertheless, this decrease was not significant (Figures 7A-C, 8A-C, and 9A-C).

## Discussion

Liver is the main immunological organ with diverse metabolic functions in homeostasis.^31-33^ Hepatocytes (liver cells) make up 70%-80% of liver volume, while the rest 20%-30% of the liver is made up of other cells.^34,35^ Mitochondria have a tubular morphology and tend to undertake 2 contrasting events of fission and fusion.^36^ Additionally, mitochondrial dynamics take part a vital role in apoptosis, and DRP1, the mitochondrial fission protein, is essential for the process fission of mitochondria. Mitochondrial damage is considered to be the main characteristic during the pathogenesis of persistent hepatitis B and C infections.^3-5^ It is well documented that HBV infection is linked with mitochondrial depolarization, defective mitochondria, ROS generation, and deregulation in calcium homeostasis. During the progression of HBV-associated liver disease hepatocellular carcinoma (HCC), mitochondria lack cristae and swollen mitochondria directly incriminate the mitochondrial injury.^6-8^ There is a dire need to investigate the mechanism by which calcium homeostasis and elevated ROS levels can be regulated in HBV infection. Thus, any interventions restoring the normal mitochondrial functions by reducing the ROS and associated damage caused by deregulation in calcium homeostasis can be beneficial for cell survival and reducing the risk of liver damage.

In this report, we explored the outcome of antioxidants (NAC) and calcium modulators EGTA-AM, BAPTA-AM, and Ru360 in the prevention of mitochondrial damage/mitophagy/fragmentation caused by HBV infection.

Clear fragmented mitochondria were observed in HepAD38 and HepG2/pHBV1.3 HBV infected cells before treatment with antioxidant (NAC). Our results indicate that NAC treatment partially reverses the normal tubular shape of mitochondria as well as reduces the mitochondrial fission markers DRP1 and p-DRP1 (Ser616) and thus has a protective role in the survival of HBV-infected cells. N*-*acetyl-l-cysteine is used in a wide range of illnesses and is also used as a dietary supplement. In a study conducted by Liu et al,^37^ NAC is reported to have hepatoprotective effect by lowering efavirenz-induced hepatotoxicity both in vitro and in vivo. N*-*acetyl-l-cysteine is reported to counteract the oxygen-free radical effects^25,26^ and improve antitumor response of interferon alpha by NF-κB downregulation in HepG2 and Huh7 liver cancer cells. These effects are more pronounced after the treatment of NAC.

Hepatitis B virus provoked the expression and phosphorylation (Ser616) of DRP1 as reported previously.^5^ Dynamin-related protein 1 has an impact on mitochondrial morphology; therefore, we used DRP1 antibody that can particularly be acquainted with phosphorylation (Ser616) residue and determined the effect of antioxidant (NAC) on Drp1-mediated mitochondrial fission and phosphorylation (Ser616) of DRP1 by treating the monolayer of HBV-expressing cells. Our results provide evidence for the significant downregulation in the expression of phosphorylation of (Ser616) DRP1 in HBV-expressing cells, treated with NAC.

Viruses utilize the universal Ca^2+^ signaling to generate a tailored cellular environment for their survival. In addition to its ability to damage the plasma membrane Ca^2+^ ATPase, the multifunctional HBx co-localized by voltage-dependent anion-selective channel protein 3 (VDAC3), is connected to apoptosis by halting ER/mitochondria coupling and stirring up cytochrome c release from mitochondria. All these mechanisms direct to an elevated level of Ca^2+^, which initiates the viral replication and core assembly.

In our study, HBV-expressing cells were treated with calcium modulators (EGTA-AM, BAPTA-AM, and Ru360). EGTA-AM and BAPTA-AM are calcium chelators and Ru360 is a calcium blocker that exclusively blocks Ca^2+^ uptakes into mitochondria. Result analysis indicates that instead of complete mitochondrial fragmentation, there was a mere swelling or partial fragmentation of mitochondria was observed in EGTA-AM- (Figure 4A and 4B), BAPTA-AM- (Figure 5A and 5B), and Ru360-treated (Figure 6A and 6B) HBV expressing cells in contrast with the untreated cells. These findings were further confirmed by the immunoblot analysis of DRP1 and p-DRP1(Ser616) where downregulation of both of these mitochondrial fission marker proteins was seen in treated as compared to untreated cells. Thus, it signifies that a partial prevention of mitochondrial fragmentation is achieved by regulating the calcium homeostasis in cell lines of HepG2/pHBV1.3 and HepAD38.

Overall the findings of the study positively designate toward positive effects of antioxidants and calcium modulators on HBV-induced mitochondrial damage, with a more pronounced effect in the case of antioxidant treatment. These significant results suggest that virus infection induced mitochondrial damage that antioxidants, calcium inhibitors, and calcium chelators can prevent. In the future, these mechanisms can be exploited for developing strategies to prevent HBV-induced liver damage. Moreover, this work provides a new avenue of therapeutics against chronic hepatitis, which often progresses to liver cancer in these patients. Further drugs as well as the drug targets against various proteins of the mitochondria and Ca^2+^ homeostatic pathways, ultimately preventing the mitochondrial damage, can be explored.

## Conclusions

The current study highlights the effect of various pharmacological reagents such as antioxidant NAC, calcium chelators (EGTA-AM, BAPTA-AM), and calcium inhibitors (Ru360) in the reduction of mitochondrial fragmentation. Cumulatively, the result of our study provides evidence for downregulating the levels of mitochondrial fission protein; however, this partial prevention was more obvious in the case of antioxidant (NAC) as compared to calcium modulators. Therefore, it is proposed that by inhibiting oxidative stress (ROS) and blocking the uptake of calcium by mitochondria, the damage could be reduced. Further work is required to determine whether this decrease in the expression of the target protein is due to the direct or indirect effect of NAC, EGTA-AM, BAPTA-AM, and Ru360 treatment. Thus, the current study, in this regard, provides a new avenue of possibility in direct therapeutic interventions regulating the mitochondrial fragmentation for HBV infection clearance/management that often progresses to liver cancer.

## Figures and Tables

**Supplementary Figure 1.**
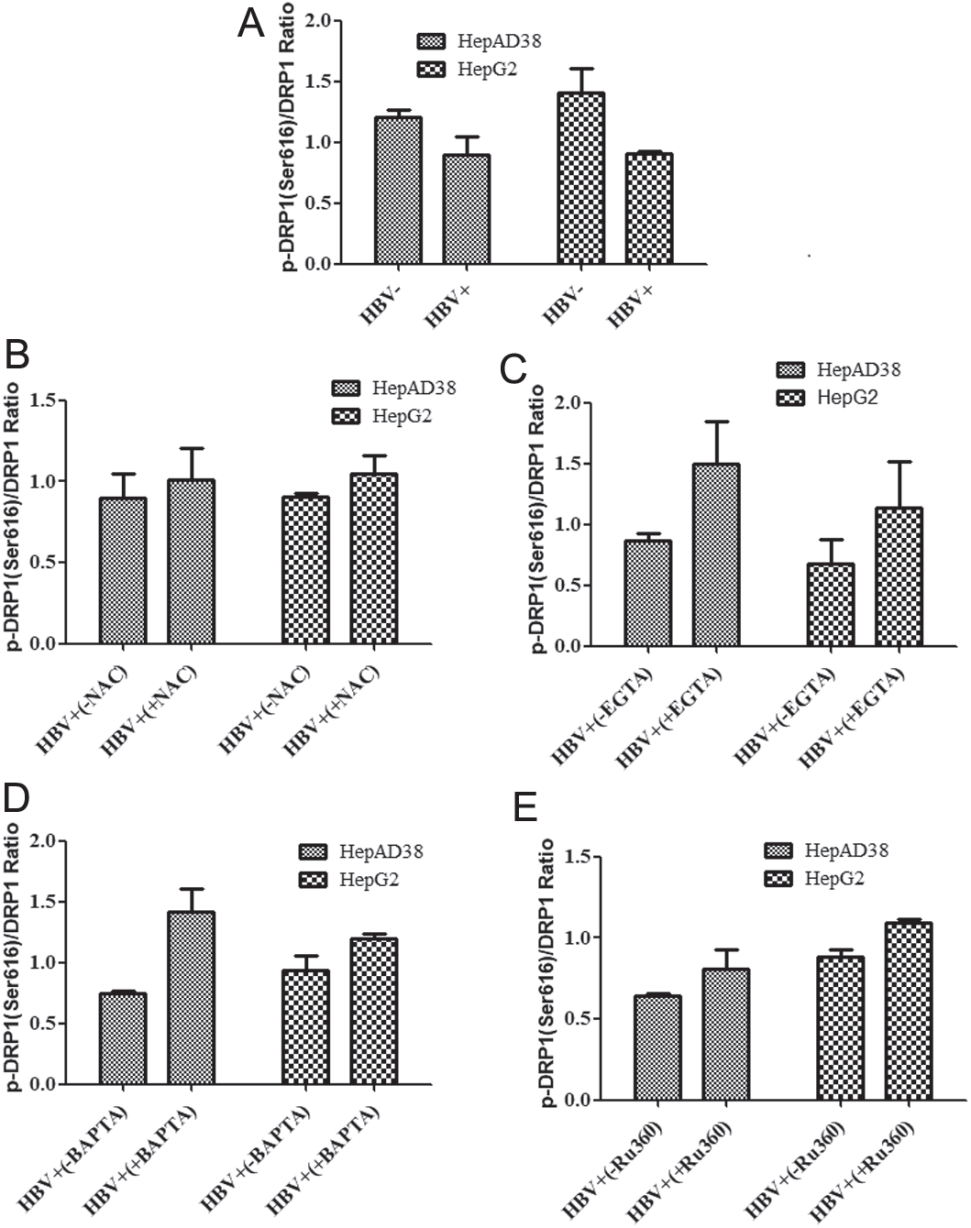
Ratio of p-DRP1/DRP1 pre and post HBV infection. (A) Ratio of p-DRP1/DRP1 was down regulated in infected cells as compared to uninfected control cells. (B-E) Ratio of p-DRP1/DRP1 was up regulated in infected cells after treatment with NAC, EGTA, BAPTA, and Ru360. A non significant trend was observed. Student’s *t-*test was applied for statistical analysis.

**Supplementary Table 1.** Protein Quantification Data Of p-DRP1 (Ser616) Protein

	HepAD38		HepG2	
	Control	Infected	Control	Infected
	HBV-	HBV+	HBV-	HBV+
	20267.2	26921.47	21306.77	41231.38
	17773.61	32846.7	17480.55	41829.02
	15990.09	37666.33	11293.52	17175.98
	8436.368	16871.46	13116.07	40873.72
	17773.61	26032.92	19808	20781.1
	5316.296	26009.39	24075.46	31525.7
	7494.205	18314.86	4942.69	17587.15
	13551.87	30605.8	12014.95	39722.09
	**HepAD38**		**HepG2**	
	**HBV+(**-**NAC)**	**HBV+(+NAC)**	**HBV+(**-**NAC)**	**HBV+(+NAC)**
Experiment 1	26921.47	15435.68	41231.38	24467.77
Experiment 2	32846.7	12217.13	41829.02	25584.36
Experiment 3	25831.46	14233.58	35374.64	25957.47
	**HepAD38**		**HepG2**	
	**HBV+(**-**EGTA)**	**HBV+(+EGTA)**	**HBV+(**-**EGTA)**	**HBV+(+EGTA)**
Experiment 1	37666.33	16371.78	17175.98	12393.35
Experiment 2	16871.46	11009.88	40873.72	19609.45
Experiment 3	25060.04	19594.73	33599.84	21426.38
	**HepAD38**		**HepG2**	
	**HBV+(-BAPTA)**	**HBV+(+BAPTA)**	**HBV+(-BAPTA)**	**HBV+(+BAPTA)**
Experiment 1	26032.92	10677.92	20781.1	12002.59
Experiment 2	26009.39	16130.8	31525.7	29299.41
Experiment 3	29691.66	28027.14	49297.95	30211.68
	**HepAD38**		**HepG2**	
	**HBV+(**-**Ru360)**	**HBV+(+Ru360)**	**HBV+(**-**Ru360)**	**HBV+(+Ru360)**
Experiment 1	18314.86	16865.52	17587.15	17843.52
Experiment 2	30605.8	26575.92	39722.09	17777.36
Experiment 3	23470.87	10284.27	41199.38	39950.75
